# Lower extremities paresis due to ossification of the Posterior Longitudinal Ligament

**Published:** 2012-11

**Authors:** Parandoush Sepehri, Elham Shobeiri

**Affiliations:** ^*a*^Department of Neurosurgery, Kermanshah University of Medical Sciences, Kermanshah, Iran.; ^*b*^Department of Radiology, Kermanshah University of Medical Sciences, Kermanshah, Iran.

## Abstract

Ossification of the Posterior Longitudinal Ligament (OPLL) is a condition caused by new bone developing in the posterior longitudinal ligament within the spinal canal. OPLL is a common cause of myelopathy in East Asian countries. Here, we report a case with OPLL in thoracic and lumbar spine in Kermanshah, West of Iran.

**Case:**

A 40 years old man gradually developed lower extremities paresis with defecation or voiding difficulties. Radiographic evaluation by computed tomography (CT) and magnetic resonance imaging (MRI) confirmed the mass of ossification at the posterior aspect of the T2-T4 and T12-L1 of vertebral body with spinal cord compression. Although OPLL is a common cause of myelopathy in the East Asian countries, it should be included in differential diagnosis of myelopathy in other regions.

**Keywords:**

Ossification of the posterior longitudinal ligament (OPLL), Spinal canal, Myelopathy


					Volume 4, Suppl. 1 Nov 2012
Publisher: Kermanshah University of Medical Sciences
URL: http://www.jivresearch.org
Copyright:
Congress of Iranian Neurosurgeons, 14-16 November 2012, Kermanshah, Iran
Paper No. 46
Lower extremities paresis due to ossification of the Posterior
Longitudinal Ligament
Parandoush Sepehri a,*
, Elham Shobeiri b
a
Department of Neurosurgery, Kermanshah University of Medical Sciences, Kermanshah, Iran.
b
Department of Radiology, Kermanshah University of Medical Sciences, Kermanshah, Iran.
Abstract:
Ossification of the Posterior Longitudinal Ligament (OPLL) is a condition caused by new bone developing in the
posterior longitudinal ligament within the spinal canal. OPLL is a common cause of myelopathy in East Asian countries.
Here, we report a case with OPLL in thoracic and lumbar spine in Kermanshah, West of Iran.
Case: A 40 years old man gradually developed lower extremities paresis with defecation or voiding difficulties.
Radiographic evaluation by computed tomography (CT) and magnetic resonance imaging (MRI) confirmed the mass
of ossification at the posterior aspect of the T2-T4 and T12-L1 of vertebral body with spinal cord compression.
Although OPLL is a common cause of myelopathy in the East Asian countries, it should be included in differential
diagnosis of myelopathy in other regions.
Keywords:
Ossification of the posterior longitudinal ligament (OPLL), Spinal canal, Myelopathy
* Corresponding Author at:
Parandoush Sepehri: MD, Department of Neurosurgery, Kermanshah University of Medical Sciences, Kermanshah, Iran.Cell phone: +989188316147,
E-mail: ps_paris@yahoo.com, (Sepehri P.).

				

